# Replicating a COVID-19 study in a national England database to assess the generalisability of research with regional electronic health record data

**DOI:** 10.1136/bmjopen-2024-093080

**Published:** 2025-04-23

**Authors:** Richard Williams, David Jenkins, Thomas Bolton, Adrian Heald, Mehrdad Mizani, Matthew Sperrin, Niels Peek, on behalf of the CVD-COVID-UK/COVID-IMPACT Consortium, Jon Boyle

**Affiliations:** 1 Division of Informatics, Imaging and Data Science, The University of Manchester, Manchester, UK; 2 NIHR Applied Research Collaboration Greater Manchester, Manchester, UK; 3 Data Science Centre, British Heart Foundation, London, UK; 4 Endocrinology and Diabetes, Salford Royal Hospitals NHS Trust, Salford, UK; 5 The School of Medicine, The University of Manchester, Manchester, UK; 6 Institute of Health Informatics, University College London, London, UK; 7 THIS Institute, University of Cambridge, Cambridge, UK

**Keywords:** Observational Study, Electronic Health Records, Retrospective Studies, DIABETES & ENDOCRINOLOGY

## Abstract

**Abstract:**

**Objectives:**

To assess the degree to which we can replicate a study between a regional and a national database of electronic health record data in the UK. The original study examined the risk factors associated with hospitalisation following COVID-19 infection in people with diabetes.

**Design:**

A replication of a retrospective cohort study.

**Setting:**

Observational electronic health record data from primary and secondary care sources in the UK. The original study used data from a large, urbanised region (Greater Manchester Care Record, Greater Manchester, UK—2.8 m patients). This replication study used a national database covering the whole of England, UK (NHS England’s Secure Data Environment service for England, accessed via the BHF Data Science Centre’s CVD-COVID-UK/COVID-IMPACT Consortium—54 m patients).

**Participants:**

Individuals with a diagnosis of type 1 diabetes or type 2 diabetes prior to a positive COVID-19 test result. The matched controls (3:1) were individuals who had a positive COVID-19 test result, but who did not have a diagnosis of diabetes on the date of their positive COVID-19 test result. Matching was done on age at COVID-19 diagnosis, sex and approximate date of COVID-19 test.

**Primary and secondary outcome measures:**

Hospitalisation within 28 days of a positive COVID-19 test.

**Results:**

We found that many of the effect sizes did not show a statistically significant difference, but that some did. Where effect sizes were statistically significant in the regional study, then they remained significant in the national study and the effect size was the same direction and of similar magnitude.

**Conclusions:**

There is some evidence that the findings from studies in smaller regional datasets can be extrapolated to a larger, national setting. However, there were some differences, and therefore replication studies remain an essential part of healthcare research.

**STRENGTHS AND LIMITATIONS OF THIS STUDY**

- The same team performed the original study and this replication study.
- The underlying data sources, while similar, had differences that may have affected the results.
- The focus of replication was a single outcome for a single condition and may not generalise to other disease areas.

## Introduction

 Observational studies using electronic health record (EHR) data are a critical component of the evidence base in population health and epidemiology. However, their findings carry less weight in evidence-based medicine when compared with more conclusive results such as those from randomised control trials. This is partly due to concerns about generalisability and the potential for confounding biases. Replication, the process of repeating a study with a different population or data source, is crucial for strengthening the evidence base in observational research. Successful replication of findings can significantly improve our confidence in their validity and generalisability, leading to a more robust foundation for policy and clinical practice decisions.

Reproducibility is one of the greatest challenges in the area of observational studies.^1 2^ Goodman *et al* define three terms for discussing research reproducibility: methods reproducibility, results reproducibility and inferential reproducibility.^3^ Methods reproducibility is the degree to which a publication includes sufficient information such that other researchers could repeat the analysis. Results reproducibility is the degree to which other researchers can achieve the same results.

We have previously published a study where we compared hospitalisation rates of patients in Greater Manchester (GM) with type 1 diabetes (T1D) or type 2 diabetes (T2D) after contracting COVID-19 when compared with age-matched and sex-matched controls.^4^ The study reported that following confirmed infection with COVID-19, a number of factors are associated with increased levels of hospitalisation in individuals with T1D and T2D. For patients with T1D, older age, increased social disadvantage, and having hypertension orchronic obstructive pulmonary disease (COPD) were associated with an increased risk of hospitalisation. Other factors were non-significant, potentially due to the small population size. Patients with T2D had the same risk factors as patients with T1D, but with the addition that male sex, non-white ethnicity and severe mental illness had an increased risk of hospitalisation, while taking metformin and low cholesterol levels were associated with a reduced risk of hospitalisation. In this study, we will attempt to replicate these findings in a national database covering the whole of England.

For this replication study, methods reproducibility should have been trivial as we performed the original analysis. However, this was not the case, and in a separate paper, we discuss the methodological factors that make replication problematic, such as differences in the governance, the data structure and the data processing.^5^ Inferential reproducibility is not possible as it is the degree to which different researchers reach the same conclusions from similar results. Therefore, in this paper, our objective is to assess the degree to which we can achieve results reproducibility between a regional and a national database of electronic health record data in the UK.

If results reproducibility can be achieved, then this will provide evidence that, under certain circumstances, scientific conclusions drawn from regional datasets can be extrapolated nationally.

## Methods

### Study design

This is a replication of a retrospective cohort study using observational EHR data from primary and secondary care sources in the UK.

### Data sources

The data for the original study were from the Greater Manchester Care Record (GMCR). The GMCR is a shared care record containing primary and secondary care data for the residents of GM. The database contains all primary care data, and all hospital admission data, for patients registered to a general practice (GP) in GM who have not opted out of data sharing.

The data for this replication study were from the NHS England National Secure Data Environment (National SDE). The National SDE provides access to a range of national datasets relating to healthcare. Data were made available for COVID-19 research through the CVD-COVID-UK/COVID-IMPACT Consortium which is coordinated by the BHF Data Science Centre and led by Health Data Research UK. The data used for this study were as follows: primary care data from the General Practice Extraction Service Data for Pandemic Planning and Research (GDPPR);^6^ secondary care data from Hospital Episode Statistics (HES) Admitted Patient Care (APC);^7^ and COVID-19 test data from the Second-Generation Surveillance System (SGSS) dataset.^8^ The differences are summarised in table 1.

**Table 1:** Differences between the original Greater Manchester study and the two replication studies

	Original study	National analysis 1	National analysis 2
Population	Patients registered with a GP in Greater Manchester. Does not include individuals who have opted out of secondary use of their GP data.	Patients registered with a GP in England, UK, in practices that opted in for GPES extraction. Does not include individuals who have opted out of secondary use of their GP data.	
Primary care data	Direct feed from GP practices. Containing all events in the patient record.	Data from the GDPPR dataset. Contains a subset of records in the patient record that were both available via GPES and considered relevant to pandemic planning and research.	
Admission data	Direct feed from each hospital within GM	HES APC data	
COVID-19 test data	From GP record	From GP record	From SGSS data and GP record

APC, Admitted Patient Care; GDPPR, General Practice Extraction Service Data for Pandemic Planning and Research; GP, General Practice; GPES, General Practice Extraction Service; HES, Hospital Episode Statistics; SGSS, Second Generation Surveillance System.

### Setting

In the original study, all patients from GM (population 2.8 m) with a positive COVID-19 test in their primary care record between 1 January 2020 (month of first UK cases of COVID-19) and 31 May 2021 were eligible.

In this replication study, we have a larger data source. Patients are now from the whole of England (population 54 m after excluding ~1.3 m opt-outs).^9^ COVID-19 tests are from the SGSS, in addition to those from the primary care record. The date range is now 1 January 2020 to 1 January 2023. The SGSS contains all community COVID-19 test results and so is more complete than the COVID-19 results that appear in a patient’s primary care record.

### Approach

We conducted two analyses. Our initial GM study relied on COVID-19 test results that appeared in the primary care record. Therefore, the first analysis was an attempt to reproduce the results of our original study, by only using the COVID-19 test data from the primary care part of the National SDE. The second analysis used the COVID-19 test data from the SGSS, in addition to the primary care data, as this is how researchers using the National SDE would obtain COVID-19 test results.

### Study population

For all analyses, the main cohort was defined as patients with a diagnosis of T1D or T2D prior to a positive COVID-19 test result. The controls were patients who had a positive COVID-19 test result, but who did not have a diagnosis of diabetes prior to the date of their positive COVID-19 test result. Each patient in the main cohort was matched with up to three controls. Controls were not reused for multiple patients. Matching was done on age at COVID-19 diagnosis, sex and approximate date (within 2 weeks either side) of COVID-19 test. The date of COVID-19 test is important as outcomes differ depending on the particular wave or variant of COVID-19 that they contracted. Further details of exactly how the cohorts were defined can be found in the original paper,^4^ and all clinical code lists and analysis code is available here: https://github.com/UoM-Data-Science-Platforms/gm-sde/tree/master/projects/020-Heald.

### Variables

The outcome is all-cause hospitalisation within 28 days of a positive COVID-19 test, or in the 2 days prior to account for people admitted to hospital due to COVID-19 but only tested once in hospital. The original study used feeds of admissions data from each hospital within GM. This replication study used the APC table from HES data.

The covariates are a subset of those from the original study. They are the following: year of birth; sex; ethnicity; deprivation via the Townsend score (a measure of social deprivation^10^); latest values prior to the COVID-19 result for body mass index (BMI), Hba1c, cholesterol (total, LDL and HDL) and eGFR; smoking status; whether the patient has COPD, asthma, a severe mental illness or hypertension; and whether the patient is currently prescribed an ACE inhibitor or ARB, aspirin, clopidogrel or metformin. The covariates in the original study that were not available for this replication study were as follows: latest values prior to COVID-19 result for vitamin D, testosterone and sex hormone-binding globulin. These biomarkers were not available in the GDPPR dataset, which contains a subset of SNOMED concepts from a patient’s primary care record, and therefore were excluded from the analysis. Testosterone and sex hormone-binding globulin had no effect in the original study, while low vitamin D had a small association with increased incidence of hospital admission.

### Statistical methods

The original study’s objective was to identify potential factors relating to an increased likelihood of hospital admission in individuals with diabetes, to assess the difference in risk between individuals with and without diabetes and to investigate if any difference in risk could be explained by routinely measured factors. The statistical analysis methods are an exact replication of the previous study.^4^ A brief overview is as follows.

Modelling was conducted using conditional logistic regression with hospitalisation within 28 days of a positive COVID-19 test as the outcome. We analysed the individuals with diabetes, without the matched controls, using a univariable logistic regression for each factor in turn, for the two groups (T1D and T2D) separately. We then fitted a multivariable model using the patients with diabetes and their controls, with diabetes diagnosis as a covariate and adjusting for sex, ethnicity, Townsend score, hypertension, COPD and BMI.

Following these analyses, we compared the national effect sizes and ORs to our previous work from the GMCR dataset. In addition to a descriptive comparison, we also calculated a conservative 95% CI for the difference between the ORs to find whether there was a statistically significant difference between the effect sizes between GM and the national data.

This analysis was performed according to a prespecified analysis plan published on GitHub, along with the phenotyping and analysis code (https://github.com/BHFDSC/CCU040_01).

### Patient and public involvement

The CVD-COVID-UK/COVID-IMPACT Approvals & Oversight Board membership includes five public contributors who ensure that the public/patient voice is considered and embedded appropriately in our projects.

The public contributors review and discuss project proposals (and research outputs) with researchers to ensure work being carried out meets the interests of people affected by heart and circulatory disease or other health conditions, to address any patient and/or public concerns and to advise on best approaches for patient and public involvement throughout the project lifecycle.

## Results

**Figure 1:**
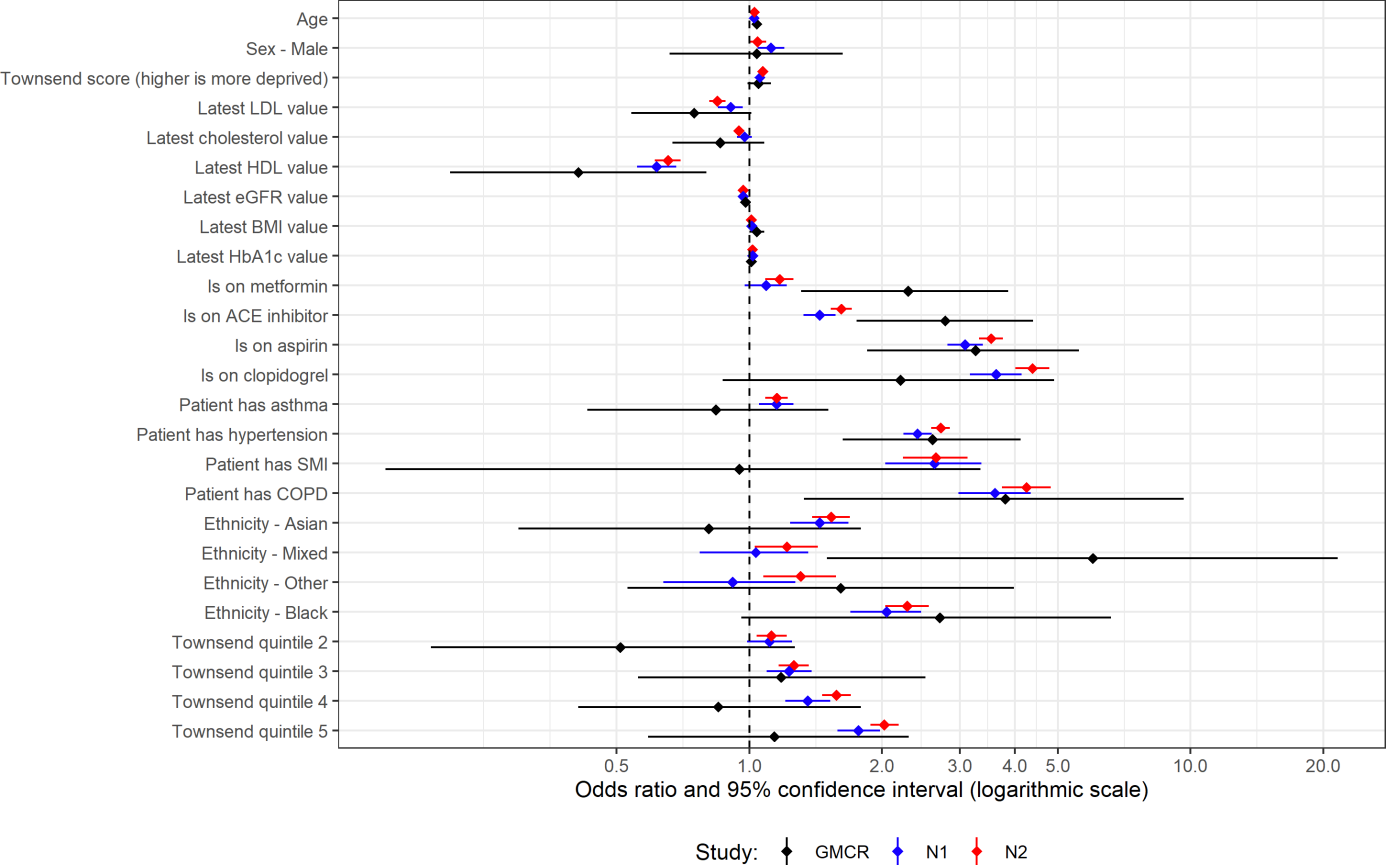
Univariable analysis for patients with type 1 diabetes. ‘GMCR’ is the original published study (Greater Manchester Care Record), ‘N1’ is the first replication analysis using COVID-19 test data from the primary care data feed and ‘N2’ is the second replication analysis using the Second-Generation Surveillance System for the COVID-19 test results.

Our objective is to demonstrate the degree to which results reproducibility can be achieved. Therefore, all ORs and CIs are displayed visually and discussed descriptively. Full tables with the numeric data for figures1^4^ are available in the supplementary material (online supplemental tables S1–S4).

**Figure 2:**
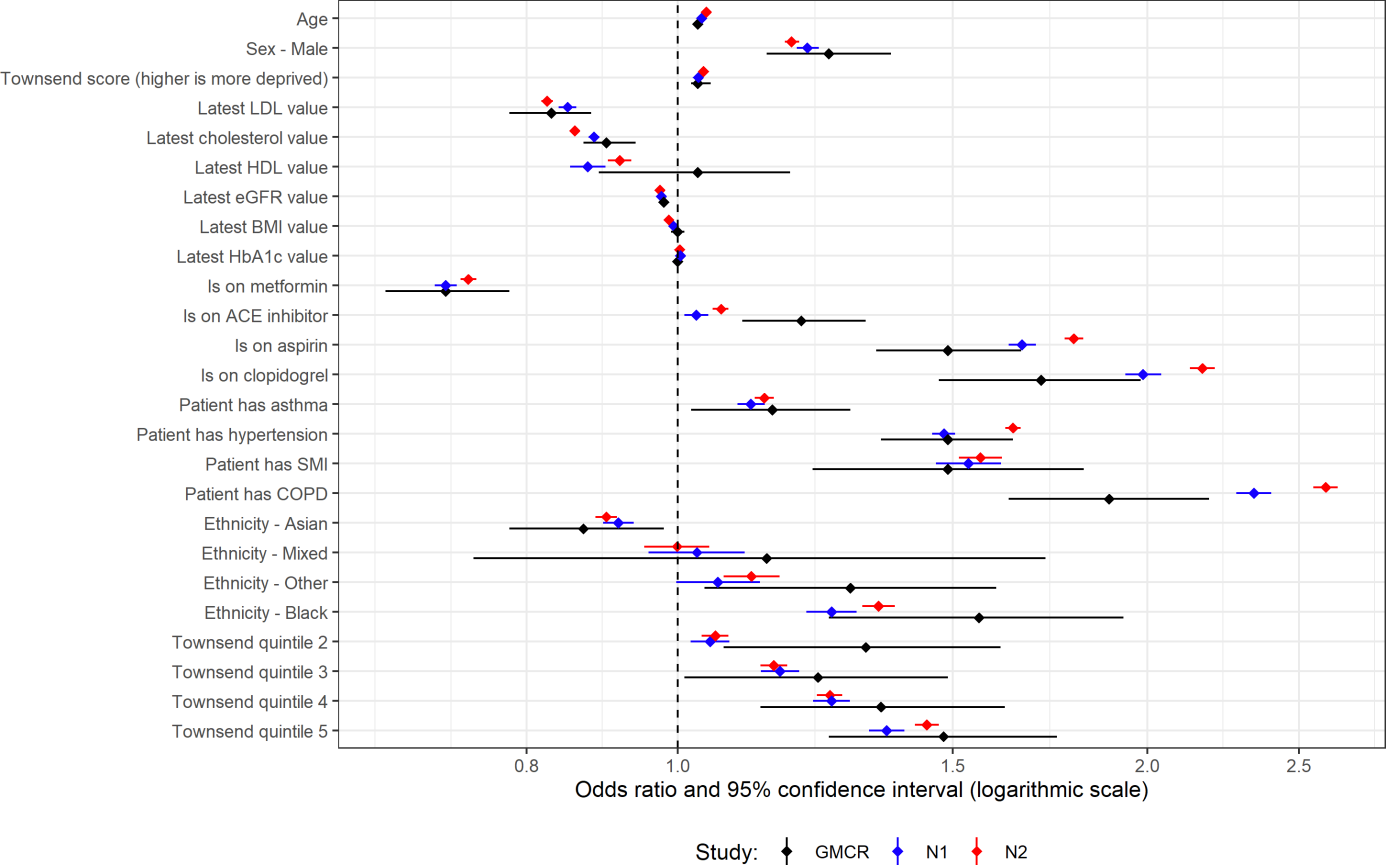
Univariable analysis for patients with type 2 diabetes. ‘GMCR’ is the original published study (Greater Manchester Care Record), ‘N1’ is the first replication analysis using COVID-19 test data from the primary care data feed and ‘N2’ is the second replication analysis using the Second-Generation Surveillance System for the COVID-19 test results.

**Figure 3:**
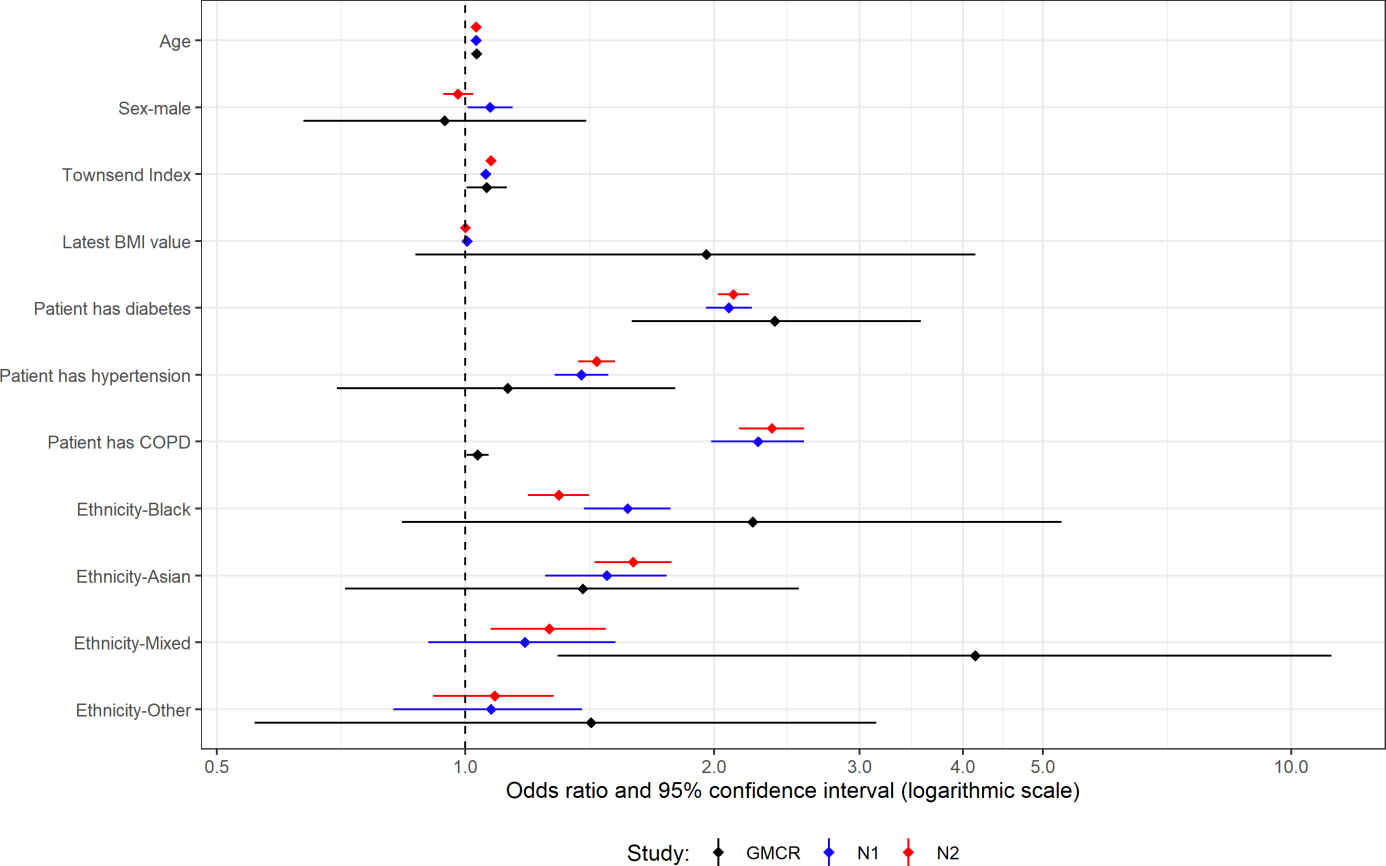
Multivariable analysis for patients with type 1 diabetes and their controls. ‘GMCR’ is the original published study (Greater Manchester Care Record), ‘N1’ is the first replication analysis using COVID-19 test data from the primary care data feed and ‘N2’ is the second replication analysis using the Second-Generation Surveillance System for the COVID-19 test results.

**Figure 4:**
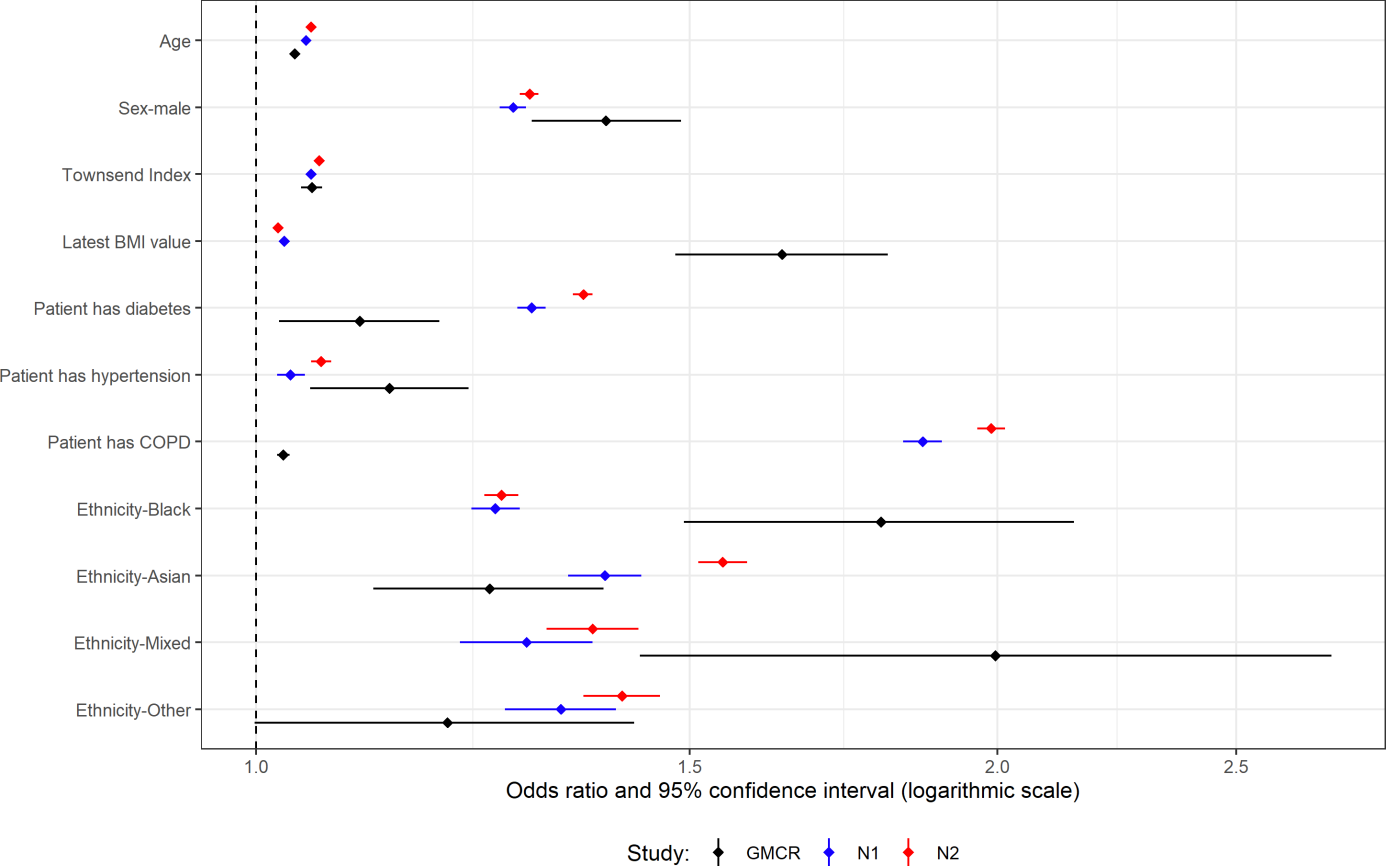
Multivariable analysis for patients with type 2 diabetes and their controls. ‘GMCR’ is the original published study (Greater Manchester Care Record), ‘N1’ is the first replication analysis using COVID-19 test data from the primary care data feed and ‘N2’ is the second replication analysis using the Second-Generation Surveillance System for the COVID-19 test results.

### Population comparison

Both national analyses benefited from a much larger population. The original GM study had 862 patients with T1D and a positive COVID-19 test result, while the first national analysis had 38 523, and the second had 77 392 (table 2). The original study had 13 225 patients with T2D and a positive COVID-19 test result, while the first national analysis had 448 829, and the second had 836 532 (table 3). We have previously published a clinical paper focussing on the individuals with T1D.^11^

**Table 2:** Characteristics of the individuals with type 1 diabetes (T1D) and their controls for the three studies

Variable	Original study		National analysis 1		National analysis 2	
	Controls	T1D	Controls	T1D	Controls	T1D
N	2573 (100%)	862 (100%)	114 790 (100%)	38 523 (100%)	223 995 (100%)	77 392 (100%)
Admission (within 28 days)	120 (5%)	86 (10%)	3735 (3%)	3422 (9%)	8665 (4%)	8263 (11%)
Age (years)	39.0 (17.0)	39.4 (17.4)	40.3 (18.0)	40.5 (18.2)	38.6 (18.3)	38.9 (18.4)
Sex (is male)	1349 (52.4%)	454 (52.7%)	58 290 (50.8%)	19 655 (51.0%)	117 304 (52.4%)	40 722 (52.6%)
Townsend score (higher is more deprived)	0.9 (3.7)	0.9 (3.6)	−0.1 (3.6)	−0.2 (3.5)	0.0 (3.6)	−0.1 (3.5)
Townsend quintile (higher is more deprived)						
1 (least deprived)	447 (17%)	135 (16%)	24 210 (21%)	7889 (21%)	46 237 (21%)	15 612 (20%)
2	364 (14%)	126 (15%)	23 891 (21%)	8335 (22%)	46 173 (21%)	16 372 (21%)
3	430 (17%)	150 (17%)	22 092 (19%)	7686 (20%)	42 955 (19%)	15 548 (20%)
4	564 (22%)	202 (23%)	21 294 (19%)	7340 (19%)	42 809 (19%)	15 136 (20%)
5 (most deprived)	768 (30%)	249 (29%)	23 303 (20%)	7273 (19%)	45 821 (21%)	14 724 (19%)
Latest BMI value	27.9 (6.2)	27.2 (5.8)	27.9 (6.7)	27.4 (6.1)	27.6 (6.8)	27.0 (6.1)
Latest LDL cholesterol value	2.9 (0.9)	2.5 (1.0)	2.9 (0.9)	2.4 (0.9)	2.9 (0.9)	2.4 (0.9)
Latest HDL cholesterol value	1.4 (0.4)	1.4 (0.4)	1.4 (0.4)	1.5 (0.5)	1.4 (0.4)	1.5 (0.4)
Latest eGFR value	82.3 (13.8)	80.5 (18.1)	81.1 (13.7)	80.3 (19.4)	80.6 (14.2)	79.7 (20.3)
Latest HbA1c value	34.5 (8.8)	67.6 (22.7)	36.5 (4.2)	66.8 (18.8)	36.4 (4.2)	67.3 (19.2)
Latest total cholesterol value	5.0 (1.0)	4.6 (1.1)	5.0 (1.1)	4.5 (1.1)	5.0 (1.1)	4.5 (1.1)
Current smoking status						
Non-smoker	1800 (70%)	593 (69%)	98 551 (86%)	32 924 (86%)	191 091 (85%)	65 661 (85%)
Smoker	773 (30%)	269 (31%)	16 239 (14%)	5599 (15%)	32 904 (15%)	11 731 (15%)
Patient has asthma	430 (17%)	149 (17%)	19 453 (17%)	6583 (17%)	35 532 (16%)	12 782 (17%)
Patient has COPD	41 (2%)	21 (2%)	1659 (1%)	599 (2%)	2940 (1%)	1112 (1%)
Patient has severe mental illness	41 (2%)	21 (2%)	921 (1%)	387 (1%)	1757 (1%)	761 (1%)
Patient has hypertension	257 (10%)	197 (23%)	11 965 (10%)	8580 (22%)	20 990 (9%)	15 869 (21%)
Patient is on ACEI	178 (7%)	210 (24%)	5590 (5%)	6805 (18%)	9716 (4%)	12 537 (16%)
Patient is on aspirin	52 (2%)	91 (11%)	2417 (2%)	3351 (9%)	4098 (2%)	6182 (8%)
Patient is on clopidogrel	27 (1%)	37 (4%)	1038 (1%)	1215 (3%)	1922 (1%)	2361 (3%)
Patient is on metformin	9 (0%)	117 (14%)	224 (0%)	4133 (11%)	347 (0%)	7418 (10%)
Hospital length of stay (days)	3.8 (8.6)	5.2 (8.2)	2.8 (8.2)	4.0 (9.2)	2.6 (9.1)	3.8 (10.3)
Ethnicity						
White	1731 (67%)	650 (75%)	95 942 (84%)	34 755 (90%)	185 136 (83%)	69 364 (90%)
Asian	334 (13%)	73 (9%)	8977 (8%)	1701 (4%)	17 233 (8%)	3373 (4%)
Black	54 (2%)	26 (3%)	3113 (3%)	869 (2%)	6649 (3%)	1950 (3%)
Mixed	46 (2%)	10 (1%)	2128 (2%)	610 (2%)	4524 (2%)	1346 (2%)
Other	91 (4%)	33 (4%)	2497 (2%)	455 (1%)	5325 (2%)	969 (1%)
Unknown	317 (12%)	70 (8%)	2133 (2%)	133 (0%)	5128 (2%)	390 (1%)

‘Original study’ is the original published study from Greater Manchester with data from 1 January 2020 up to 31 May 2021. ‘National analysis 1’ is the first replication analysis using COVID-19 test data from the primary care data feed. ‘National analysis 2’ is the second replication analysis using the Second-Generation Surveillance System for the COVID-19 test results. The national analysis was on data from 1 January 2020 to 31 December 2022. Values are presented as either ‘mean (SD)’ or ‘count (percentage)’.

**Table 3:** Characteristics of the individuals with type 2 diabetes (T2D) and their controls for the three studies

Variable	Original study		National analysis 1		National analysis 2	
	Controls	T2D	Controls	T2D	Controls	T2D
N	37 979 (100%)	13 225 (100%)	1 298 984 (100%)	448 829 (100%)	2 354 775 (100%)	836 532 (100%)
Admission (within 28 days)	4407 (12%)	2160 (16%)	116 443 (9%)	68 659 (15%)	254 496 (11%)	155 796 (19%)
Age	62.2 (14.4)	63.1 (14.4)	62.8 (14.7)	63.3 (14.7)	63.0 (14.8)	63.5 (14.9)
Sex (is male)	20 688 (54.5%)	7427 (56.2%)	675 455 (52%)	238 400 (53%)	1 257 080 (53.4%)	454 235 (54.3%)
Townsend score (higher is more deprived)	0.4 (3.7)	1.8 (3.7)	−0.6 (3.4)	0.5 (3.7)	−0.6 (3.3)	0.6 (3.7)
Townsend quintile (higher is more deprived)						
1	7540 (20%)	1534 (12%)	325 211 (25%)	75 668 (17%)	583 601 (25%)	137 328 (16%)
2	6126 (16%)	1491 (11%)	301 249 (23%)	83 326 (19%)	546 987 (23%)	153 864 (18%)
3	6888 (18%)	2076 (16%)	253 188 (20%)	84 480 (19%)	464 107 (20%)	158 645 (19%)
4	8062 (21%)	2996 (23%)	219 340 (17%)	91 425 (20%)	404 138 (17%)	174 275 (21%)
5	9363 (25%)	5128 (39%)	199 996 (15%)	113 930 (25%)	355 942 (15%)	212 420 (25%)
Latest BMI value	28.6 (6.1)	31.7 (6.9)	28.1 (6.2)	31.9 (7.2)	28.0 (6.1)	31.7 (7.2)
Latest LDL cholesterol value	2.8 (1.0)	2.2 (0.9)	2.7 (1.0)	2.2 (0.9)	2.7 (1.0)	2.2 (1.0)
Latest HDL cholesterol value	1.4 (0.4)	1.2 (0.3)	1.5 (0.4)	1.2 (0.3)	1.5 (0.4)	1.2 (0.3)
Latest eGFR value	75.9 (15.7)	75.3 (18.7)	74.0 (16.0)	73.5 (19.2)	73.2 (16.3)	72.5 (19.8)
Latest HbA1c value	36.1 (9.1)	56.6 (21.0)	38.0 (4.1)	58.1 (17.5)	38.0 (4.2)	58.3 (17.6)
Latest total cholesterol value	4.9 (1.1)	4.3 (1.2)	4.9 (1.1)	4.3 (1.2)	4.9 (1.1)	4.3 (1.2)
Current smoking status						
Non-smoker	22 519 (59%)	7774 (59%)	1 137 301 (88%)	390 957 (87%)	2 044 839 (87%)	722 813 (86%)
Smoker	15 460 (41%)	5451 (41%)	161 683 (12%)	57 872 (13%)	309 936 (13%)	113 719 (14%)
Patient has asthma	5867 (15%)	2401 (18%)	199 605 (15%)	85 642 (19%)	345 564 (15%)	153 313 (18%)
Patient has COPD	2566 (7%)	1011 (8%)	67 251 (5%)	31 576 (7%)	123 297 (5%)	59 235 (7%)
Patient has severe mental illness	1300 (3%)	603 (5%)	15 902 (1%)	10 232 (2%)	29 230 (1%)	20 144 (2%)
Patient has hypertension	11 337 (30%)	7380 (56%)	392 765 (30%)	252 621 (56%)	714 311 (30%)	472 596 (57%)
Patient is on ACEI	7695 (20%)	6537 (49%)	165 378 (13%)	149 107 (33%)	298 067 (13%)	275 760 (33%)
Patient is on aspirin	3079 (8%)	2559 (19%)	90 899 (7%)	72 149 (16%)	165 549 (7%)	135 184 (16%)
Patient is on clopidogrel	1607 (4%)	1042 (8%)	48 941 (4%)	31 870 (7%)	89 827 (4%)	60 763 (7%)
Patient is on metformin	253 (1%)	8150 (62%)	1425 (0%)	270 421 (60%)	2632 (0%)	496 184 (59%)
Hospital length of stay (days)	6.7 (13.2)	8.2 (16.2)	5.3 (11.1)	6.4 (12.0)	5.1 (11.3)	6.2 (12.4)
Ethnicity						
White	29 405 (77%)	7981 (60%)	1 157 194 (89%)	340 211 (76%)	2 093 541 (89%)	632 016 (76%)
Asian	3082 (8%)	3274 (25%)	67 877 (5%)	73 277 (16%)	115 543 (5%)	133 818 (16%)
Black	833 (2%)	498 (4%)	27 301 (2%)	19 576 (4%)	51 998 (2%)	39 550 (5%)
Mixed	279 (1%)	148 (1%)	11 411 (1%)	5836 (1%)	21 204 (1%)	11 180 (1%)
Other	1101 (3%)	541 (4%)	18 073 (1%)	7636 (2%)	32 759 (1%)	14 335 (2%)
Unknown	3279 (9%)	783 (6%)	17 128 (1%)	2293 (1%)	39 730 (2%)	5633 (1%)

‘Original study’ is the original published study from Greater Manchester with data from 1 January 2020 up to 31 May 2021. ‘National analysis 1’ is the first replication analysis using COVID-19 test data from the primary care data feed. ‘National analysis 2’ is the second replication analysis using the Second-Generation Surveillance System for the COVID-19 test results. The national analysis was on data from 1 January 2020 to 31 December 2022. Values are presented as either ‘mean (SD)’ or ‘count (percentage)’.

Most factors analysed were comparable with a few exceptions. Smoking status was much lower nationally (14–15% vs 30–31% for T1D, 12–14% vs 41% for T2D), but this was due to a categorisation error in the original study where anyone with a history of smoking was counted as a smoker. GM is more ethnically diverse, but the GM data also has a higher proportion of unknown ethnicities, possibly because in the National SDE there are more sources of demographic data from which to determine an individuals’ ethnicity. Finally, patients in the national analyses had, on average, shorter lengths of stay in hospital. This is likely due to the later cut-off date for the national analyses, where the combined effect of the reduced severity of later strains and the vaccination programme mean that later diagnoses of COVID-19 are less likely to be severe.

### T1D univariable analyses

Out of 25 variables analysed, only three (ACE inhibitor, metformin or mixed ethnicity) showed a statistically significant difference in effect size between GM and the national data (online supplemental table S5). Mixed ethnicity had extremely small numbers in the GM study so the discrepancy here is likely due to random chance and the inconsistencies in reporting mixed ethnicity. For prescribed medications, it is possible that not all metformin or ACE inhibitor SNOMED codes are extracted in the GDPPR dataset which may explain this discrepancy.

All variables that had statistically significant effect sizes in the original study had the same positive or negative association (OR direction) with the outcome in both national analyses (figure 1).

### T2D univariable analyses

For the first national analysis, out of 25 variables analysed, only four (latest HDL, COPD, ACE inhibitor, Townsend quintile 2) showed a statistically significant difference in effect size between GM and the national data (online supplemental table S5). For the second national analysis, there were eight that showed a difference (age, cholesterol, eGFR, COPD, ACE inhibitor, clopidogrel, aspirin, Townsend quintile 2).

All variables with statistically significant effect sizes in the original study had the same positive or negative association with the outcome in both national analyses (figure 2).

### T1D multivariable analyses

History of COPD and mixed ethnicity were the only variables with a statistically significant difference in effect size between GM and the national data (online supplemental table S6). As mentioned earlier, the original study had very few patients coded as mixed ethnicity and so had a wide CI, and while the ORs do not fall within the original CI, the CIs do overlap (figure 3).

### T2D multivariable analyses

For the first national analysis, 8 (out of 11), and for the second, 6 (out of 11) variables showed a statistically significant difference in effect size between GM and the national data (online supplemental table S6).

Most variables have an OR in the national analyses that is outside the CI of the original study (figure 4). However, all ORs are in the same direction as in the original study. Age, Townsend index and hypertension all have a small, but significant, effect size in all three analyses. Being male, or non-white ethnicity, has large effect sizes in all three analyses, though black ethnicity has a smaller OR in the national analyses (first national OR=1.25 and second national OR=1.26 vs GM OR=1.79). Patients with diabetes and patients with COPD have a much larger OR in the national analyses (diabetes: 1.29 and 1.36 vs 1.1, COPD: 1.87 and 1.99 vs 1.03). Latest BMI has much smaller ORs in the national analyses (BMI: 1.03 and 1.02 vs 1.64).

## Discussion

We have conducted a study to determine the extent to which results replicate between a regional and a national database of electronic healthcare record data.

EHR data can be variable in quality and contain many unknowns and challenges.^12^ However, they are typically analysed in large quantities which to some extent mitigates the effects of missingness and noise from random bias. Our analysis has shown that, while the actual ORs from multiple studies may vary, the direction and approximate magnitude of the effect size remain the same. All variables with a statistically significant effect size in the original analysis remained significant, and therefore, clinical decisions made on the results in the regional study would be consistent with the national analyses. This provides some evidence that the findings of regional studies can be extrapolated to a national setting.

However, there were also discrepancies, particularly in the multivariable analysis of patients with T2D and their controls. The large effect size of BMI in the original studies was much lower in the national analyses, and the effect of a patient having diabetes or COPD was much higher in the national analyses. The differences may be due to the underlying data sources, or to differences in the phenotypes as in the GM data the clinical coding was a mixture of Read v2, CTV3 and EMIS codes, while the national database was SNOMED. Therefore, it is important to replicate observational studies in different datasets to better understand the results due to genuine differences between the populations rather than those that are artefacts of the data.

The data analysis code was identical in all studies, but the data curation code was different due to differences in the underlying data. It is therefore possible that differences or mistakes in the data curation code explain some of the discrepancies. All codes used in this analysis are publicly available and therefore open to scrutiny, but it is time consuming for third-party researchers to review this code. In theory, the public nature of the code allows other researchers to identify bugs, but in practice, it is unlikely to occur. One option to discover such errors is for an independent study team to perform the same analysis on the same data. Reproduction of studies using the same data, but performed by a different study team, would be beneficial. However, even that is not a panacea, as discovered in a recent study where 174 independent teams were given the same data and the same research question, yet there was substantial heterogeneity among findings with some showing results with opposite associations with the outcome variable.^13^

The cohort in the second national analysis was approximately double the cohort for the first national analysis for both T1D (77 392 patients vs 38 523) and T2D (836 532 vs 448 829). The difference between these cohorts was the addition of the SGSS dataset to identify more COVID-19 positive tests. SGSS is a much better source of COVID-19 test data; however, there is no real difference between the results in the two national analyses, suggesting that COVID-19 tests in the primary care record are sufficient for most research.

The original study population appears to have a higher proportion of severe mental illness (SMI) when compared with the national population. The prevalence in GM is likely to be higher than that observed nationally due to the above average levels of deprivation.^14^ However, in this case, it is predominantly because not all clinical codes used in the original analysis to define SMI were available in the GDPPR dataset and so the apparent prevalence was lower nationally. The original study also had a much higher proportion of smokers. However, this was due to an error where patients who had ever had a current-smoker clinical code in their record were counted as smokers, even if they subsequently had quit. Smoking was therefore excluded from the replication study.

### Strengths and limitations

- Despite differences in the data sources, the results were remarkably similar, giving strength to the findings in both studies.
- The findings in this replication study for this particular disorder may not be transferable to other conditions, although it is likely to be similar for other long-term conditions diagnosed in primary care.
- The same researchers conducted both studies and so may have made the same conceptual or procedural errors in both studies.
- Knowing the previous study’s results may have subconsciously led us to confirm the previous findings rather than attempt to challenge them.
- The replication benefitted from a mix of original researchers and new colleagues from the national SDE, which ensured the replication was as objective as possible.

### Conclusion

In two replication studies, performed in a national database, we have shown similar results with a previous study in a smaller, regional database. This provides evidence that results in regional databases can be extrapolated to national settings. However, there were still differences, which further highlights the need for replication of observational studies using electronic health record data, and for different study teams to reproduce work using the same data.

## Supplementary material

10.1136/bmjopen-2024-093080 online supplemental file 1

## Data Availability

Data may be obtained from a third party and are not publicly available.
