# Supplementary material for: Replicating a COVID-19 study in a national England database to assess the generalisability of research with regional electronic health record data
Source: BMJ Open. 2025 Apr 23;15(4):e093080. doi: 10.1136/bmjopen-2024-093080 (PMC12020769; doi:10.1136/bmjopen-2024-093080)
Supplement: online supplemental file 1 [file bmjopen-15-4-s001.docx]

Table S1 - Univariable analysis for patients with type 1 diabetes. “GMCR” is the original published study (Greater Manchester Care Record), “N1” is the first replication study using COVID test data from the primary care data feed, and “N2” is the second replication study utilising the Second-Generation Surveillance System for the COVID test results.

|  | **GMCR** | | | **N1** | | | **N2** | | |
| --- | --- | --- | --- | --- | --- | --- | --- | --- | --- |
| **VARIABLE** | **Odds ratio** | **Lower CI** | **Upper CI** | **Odds ratio** | **Lower CI** | **Upper CI** | **Odds ratio** | **Lower CI** | **Upper CI** |
| **Age** | 1.04 | 1.02 | 1.05 | 1.03 | 1.02 | 1.03 | 1.03 | 1.03 | 1.03 |
| **Sex - Male** | 1.04 | 0.66 | 1.63 | 1.12 | 1.04 | 1.20 | 1.04 | 1.00 | 1.09 |
| **Townsend score (higher = more deprived)** | 1.05 | 0.99 | 1.12 | 1.06 | 1.05 | 1.07 | 1.07 | 1.07 | 1.08 |
| **Latest BMI value** | 1.04 | 1.00 | 1.08 | 1.01 | 1.01 | 1.02 | 1.01 | 1.01 | 1.02 |
| **Latest HbA1c value** | 1.01 | 1.00 | 1.02 | 1.02 | 1.02 | 1.02 | 1.02 | 1.02 | 1.02 |
| **Latest cholesterol value** | 0.86 | 0.67 | 1.08 | 0.98 | 0.94 | 1.01 | 0.95 | 0.92 | 0.97 |
| **Latest LDL value** | 0.75 | 0.54 | 1.01 | 0.91 | 0.85 | 0.97 | 0.85 | 0.81 | 0.88 |
| **Latest HDL value** | 0.41 | 0.21 | 0.80 | 0.62 | 0.56 | 0.68 | 0.65 | 0.61 | 0.70 |
| **Latest eGFR value** | 0.98 | 0.97 | 0.99 | 0.97 | 0.97 | 0.97 | 0.97 | 0.97 | 0.97 |
| **Patient has COPD** | 3.80 | 1.33 | 9.65 | 3.60 | 2.98 | 4.34 | 4.25 | 3.74 | 4.82 |
| **Patient has asthma** | 0.84 | 0.43 | 1.51 | 1.15 | 1.05 | 1.26 | 1.15 | 1.09 | 1.22 |
| **Patient has SMI** | 0.95 | 0.15 | 3.34 | 2.63 | 2.03 | 3.36 | 2.65 | 2.23 | 3.13 |
| **Is on ACE inhibitor** | 2.78 | 1.75 | 4.39 | 1.44 | 1.33 | 1.57 | 1.62 | 1.53 | 1.71 |
| **Is on aspirin** | 3.26 | 1.85 | 5.58 | 3.09 | 2.81 | 3.38 | 3.53 | 3.32 | 3.76 |
| **Is on clopidogrel** | 2.20 | 0.87 | 4.91 | 3.63 | 3.16 | 4.14 | 4.38 | 4.01 | 4.79 |
| **Is on metformin** | 2.29 | 1.31 | 3.86 | 1.09 | 0.98 | 1.22 | 1.17 | 1.09 | 1.26 |
| **Patient has hypertension** | 2.60 | 1.63 | 4.12 | 2.41 | 2.23 | 2.59 | 2.71 | 2.59 | 2.85 |
| **Townsend quintile 2** | 0.51 | 0.19 | 1.27 | 1.11 | 0.99 | 1.25 | 1.12 | 1.04 | 1.22 |
| **Townsend quintile 3** | 1.18 | 0.56 | 2.51 | 1.23 | 1.10 | 1.39 | 1.26 | 1.17 | 1.36 |
| **Townsend quintile 4** | 0.85 | 0.41 | 1.79 | 1.36 | 1.21 | 1.52 | 1.58 | 1.46 | 1.70 |
| **Townsend quintile 5** | 1.14 | 0.59 | 2.30 | 1.77 | 1.58 | 1.98 | 2.02 | 1.88 | 2.18 |
| **Ethnicity - Black** | 2.70 | 0.96 | 6.60 | 2.05 | 1.70 | 2.45 | 2.28 | 2.03 | 2.55 |
| **Ethnicity - Asian** | 0.81 | 0.30 | 1.79 | 1.44 | 1.24 | 1.68 | 1.53 | 1.39 | 1.69 |
| **Ethnicity - Mixed** | 6.00 | 1.50 | 21.55 | 1.04 | 0.77 | 1.36 | 1.22 | 1.03 | 1.43 |
| **Ethnicity - Other** | 1.61 | 0.53 | 3.98 | 0.92 | 0.64 | 1.27 | 1.31 | 1.08 | 1.57 |

Table S2 - Univariable analysis for patients with type 2 diabetes. “GMCR” is the original published study (Greater Manchester Care Record), “N1” is the first replication study using COVID test data from the primary care data feed, and “N2” is the second replication study utilising the Second-Generation Surveillance System for the COVID test results.

|  | **GMCR** | | | **N1** | | | **N2** | | |
| --- | --- | --- | --- | --- | --- | --- | --- | --- | --- |
| **VARIABLE** | **Odds ratio** | **Lower CI** | **Upper CI** | **Odds ratio** | **Lower CI** | **Upper CI** | **Odds ratio** | **Lower CI** | **Upper CI** |
| **Age** | 1.03 | 1.03 | 1.03 | 1.04 | 1.04 | 1.04 | 1.04 | 1.04 | 1.04 |
| **Sex - Male** | 1.25 | 1.14 | 1.37 | 1.21 | 1.19 | 1.23 | 1.18 | 1.17 | 1.20 |
| **Townsend score (higher = more deprived)** | 1.03 | 1.02 | 1.05 | 1.03 | 1.03 | 1.03 | 1.04 | 1.04 | 1.04 |
| **Latest BMI value** | 1.00 | 0.99 | 1.01 | 0.99 | 0.99 | 1.00 | 0.99 | 0.99 | 0.99 |
| **Latest HbA1c value** | 1.00 | 1.00 | 1.00 | 1.00 | 1.00 | 1.00 | 1.00 | 1.00 | 1.00 |
| **Latest cholesterol value** | 0.90 | 0.87 | 0.94 | 0.88 | 0.88 | 0.89 | 0.86 | 0.85 | 0.86 |
| **Latest LDL value** | 0.83 | 0.78 | 0.88 | 0.85 | 0.84 | 0.86 | 0.83 | 0.82 | 0.83 |
| **Latest HDL value** | 1.03 | 0.89 | 1.18 | 0.88 | 0.85 | 0.90 | 0.92 | 0.90 | 0.93 |
| **Latest eGFR value** | 0.98 | 0.98 | 0.98 | 0.98 | 0.98 | 0.98 | 0.97 | 0.97 | 0.97 |
| **Patient has COPD** | 1.89 | 1.63 | 2.19 | 2.34 | 2.28 | 2.40 | 2.60 | 2.56 | 2.65 |
| **Patient has asthma** | 1.15 | 1.02 | 1.29 | 1.11 | 1.09 | 1.14 | 1.14 | 1.12 | 1.15 |
| **Patient has SMI** | 1.49 | 1.22 | 1.82 | 1.54 | 1.46 | 1.61 | 1.56 | 1.51 | 1.61 |
| **Is on ACE inhibitor** | 1.20 | 1.10 | 1.32 | 1.03 | 1.01 | 1.05 | 1.07 | 1.05 | 1.08 |
| **Is on aspirin** | 1.49 | 1.34 | 1.66 | 1.66 | 1.63 | 1.70 | 1.79 | 1.77 | 1.82 |
| **Is on clopidogrel** | 1.71 | 1.47 | 1.98 | 1.99 | 1.94 | 2.04 | 2.17 | 2.13 | 2.21 |
| **Is on metformin** | 0.71 | 0.65 | 0.78 | 0.71 | 0.70 | 0.72 | 0.73 | 0.73 | 0.74 |
| **Patient has hypertension** | 1.49 | 1.35 | 1.64 | 1.48 | 1.46 | 1.51 | 1.64 | 1.62 | 1.66 |
| **Townsend quintile 2** | 1.32 | 1.07 | 1.61 | 1.05 | 1.02 | 1.08 | 1.06 | 1.04 | 1.08 |
| **Townsend quintile 3** | 1.23 | 1.01 | 1.49 | 1.16 | 1.13 | 1.20 | 1.15 | 1.13 | 1.18 |
| **Townsend quintile 4** | 1.35 | 1.13 | 1.62 | 1.26 | 1.22 | 1.29 | 1.25 | 1.23 | 1.28 |
| **Townsend quintile 5** | 1.48 | 1.25 | 1.75 | 1.36 | 1.33 | 1.40 | 1.44 | 1.42 | 1.47 |
| **Ethnicity - Black** | 1.56 | 1.25 | 1.93 | 1.26 | 1.21 | 1.30 | 1.35 | 1.31 | 1.38 |
| **Ethnicity - Asian** | 0.87 | 0.78 | 0.98 | 0.92 | 0.90 | 0.94 | 0.90 | 0.89 | 0.91 |
| **Ethnicity - Mixed** | 1.14 | 0.74 | 1.72 | 1.03 | 0.96 | 1.10 | 1.00 | 0.95 | 1.05 |
| **Ethnicity - Other** | 1.29 | 1.04 | 1.60 | 1.06 | 1.00 | 1.13 | 1.12 | 1.07 | 1.16 |

Table S3 - Multivariable analysis for patients with type 1 diabetes and their controls. “GMCR” is the original published study (Greater Manchester Care Record), “N1” is the first replication study using COVID test data from the primary care data feed, and “N2” is the second replication study utilising the Second-Generation Surveillance System for the COVID test results.

|  | **GMCR** | | | **N1** | | | **N2** | | |
| --- | --- | --- | --- | --- | --- | --- | --- | --- | --- |
| **VARIABLE** | **Odds ratio** | **Lower CI** | **Upper CI** | **Odds ratio** | **Lower CI** | **Upper CI** | **Odds ratio** | **Lower CI** | **Upper CI** |
| **Age** | 1.03 | 1.02 | 1.05 | 1.03 | 1.03 | 1.03 | 1.03 | 1.03 | 1.03 |
| **Townsend Index** | 1.06 | 1.00 | 1.12 | 1.06 | 1.05 | 1.07 | 1.07 | 1.07 | 1.08 |
| **Sex-male** | 0.94 | 0.64 | 1.40 | 1.07 | 1.01 | 1.14 | 0.98 | 0.94 | 1.02 |
| **Patient has diabetes** | 2.37 | 1.59 | 3.56 | 2.08 | 1.96 | 2.22 | 2.11 | 2.02 | 2.20 |
| **Patient has COPD** | 1.04 | 1.00 | 1.07 | 2.26 | 1.98 | 2.57 | 2.35 | 2.15 | 2.57 |
| **Latest BMI value** | 1.96 | 0.87 | 4.14 | 1.01 | 1.00 | 1.01 | 1.00 | 1.00 | 1.00 |
| **Ethnicity-Black** | 2.23 | 0.84 | 5.27 | 1.57 | 1.39 | 1.77 | 1.30 | 1.19 | 1.41 |
| **Ethnicity-Asian** | 1.39 | 0.72 | 2.53 | 1.48 | 1.25 | 1.75 | 1.60 | 1.43 | 1.78 |
| **Ethnicity-Mixed** | 4.15 | 1.29 | 11.18 | 1.18 | 0.90 | 1.52 | 1.26 | 1.07 | 1.48 |
| **Ethnicity-Other** | 1.42 | 0.56 | 3.14 | 1.07 | 0.82 | 1.38 | 1.09 | 0.91 | 1.28 |
| **Patient has hypertension** | 1.13 | 0.70 | 1.80 | 1.38 | 1.28 | 1.49 | 1.44 | 1.37 | 1.52 |

Table S4 - Multivariable analysis for patients with type 2 diabetes and their controls. “GMCR” is the original published study (Greater Manchester Care Record), “N1” is the first replication study using COVID test data from the primary care data feed, and “N2” is the second replication study utilising the Second-Generation Surveillance System for the COVID test results.

|  | **GMCR** | | | **N1** | | | **N2** | | |
| --- | --- | --- | --- | --- | --- | --- | --- | --- | --- |
| **VARIABLE** | **Odds ratio** | **Lower CI** | **Upper CI** | **Odds ratio** | **Lower CI** | **Upper CI** | **Odds ratio** | **Lower CI** | **Upper CI** |
| **Age** | 1.04 | 1.03 | 1.04 | 1.05 | 1.05 | 1.05 | 1.05 | 1.05 | 1.05 |
| **Townsend Index** | 1.05 | 1.04 | 1.06 | 1.05 | 1.05 | 1.06 | 1.06 | 1.06 | 1.06 |
| **Sex-male** | 1.39 | 1.29 | 1.49 | 1.27 | 1.26 | 1.29 | 1.29 | 1.28 | 1.30 |
| **Patient has diabetes** | 1.10 | 1.02 | 1.19 | 1.29 | 1.28 | 1.31 | 1.36 | 1.35 | 1.37 |
| **Patient has COPD** | 1.03 | 1.02 | 1.03 | 1.87 | 1.83 | 1.90 | 1.99 | 1.96 | 2.01 |
| **Latest BMI value** | 1.64 | 1.48 | 1.81 | 1.03 | 1.03 | 1.03 | 1.02 | 1.02 | 1.02 |
| **Ethnicity-Black** | 1.79 | 1.49 | 2.15 | 1.25 | 1.22 | 1.28 | 1.26 | 1.24 | 1.28 |
| **Ethnicity-Asian** | 1.24 | 1.12 | 1.38 | 1.39 | 1.34 | 1.43 | 1.55 | 1.51 | 1.58 |
| **Ethnicity-Mixed** | 2.00 | 1.43 | 2.73 | 1.29 | 1.21 | 1.37 | 1.37 | 1.31 | 1.43 |
| **Ethnicity-Other** | 1.20 | 1.00 | 1.42 | 1.33 | 1.26 | 1.40 | 1.41 | 1.36 | 1.46 |
| **Patient has hypertension** | 1.13 | 1.05 | 1.22 | 1.03 | 1.02 | 1.05 | 1.06 | 1.05 | 1.07 |

Table S5– Conservative 95% confidence intervals for the difference in effects between the national studies and the original study in the univariable analyses. “GMCR” is the original published study (Greater Manchester Care Record), “N1” is the first replication study using COVID test data from the primary care data feed, and “N2” is the second replication study utilising the Second-Generation Surveillance System for the COVID test results. “T1D” are the patients with type 1 diabetes and T2D are patients with type 2 diabetes. “Significant” is “Yes” if the confidence interval does not span 0 and therefore the difference effect size is statistically significant between GMCR and the national study.

| **VARIABLE** | **T1D – N1** | | **T1D – N2** | | **T2D – N1** | | **T2D – N2** | |
| --- | --- | --- | --- | --- | --- | --- | --- | --- |
|  | **CI** | **Significant** | **CI** | **Significant** | **CI** | **Significant** | **CI** | **Significant** |
| **Age** | [ 0.00, 0.03] |  | [ 0.00, 0.03] |  | [-0.01, 0.00] |  | [-0.02, -0.01] | Yes |
| **Sex** | [-0.53, 0.38] |  | [-0.46, 0.45] |  | [-0.06, 0.13] |  | [-0.04, 0.15] |  |
| **Townsend score (higher is more deprived)** | [-0.07, 0.06] |  | [-0.08, 0.04] |  | [-0.02, 0.01] |  | [-0.02, 0.01] |  |
| **Latest BMI value** | [-0.01, 0.07] |  | [-0.01, 0.07] |  | [ 0.00, 0.02] |  | [ 0.00, 0.02] |  |
| **Latest HbA1c value** | [-0.02, 0.00] |  | [-0.02, 0.00] |  | [ 0.00, 0.00] |  | [ 0.00, 0.00] |  |
| **Latest cholesterol value** | [-0.37, 0.12] |  | [-0.34, 0.14] |  | [-0.02, 0.06] |  | [ 0.01, 0.09] | Yes |
| **Latest LDL value** | [-0.51, 0.13] |  | [-0.44, 0.19] |  | [-0.09, 0.04] |  | [-0.05, 0.07] |  |
| **Latest HDL value** | [-1.09, 0.27] |  | [-1.14, 0.21] |  | [ 0.02, 0.31] | Yes | [-0.03, 0.26] |  |
| **Latest eGFR value** | [ 0.00, 0.02] |  | [ 0.00, 0.02] |  | [ 0.00, 0.00] |  | [ 0.01, 0.01] | Yes |
| **Patient has COPD** | [-0.96, 1.06] |  | [-1.11, 0.89] |  | [-0.36, -0.06] | Yes | [-0.47, -0.17] | Yes |
| **Patient has asthma** | [-0.95, 0.32] |  | [-0.95, 0.31] |  | [-0.09, 0.15] |  | [-0.11, 0.13] |  |
| **Patient has SMI** | [-2.59, 0.55] |  | [-2.59, 0.54] |  | [-0.24, 0.18] |  | [-0.25, 0.15] |  |
| **Is on ACE inhibitor** | [ 0.19, 1.12] | Yes | [ 0.08, 1.01] | Yes | [ 0.06, 0.25] | Yes | [ 0.03, 0.21] | Yes |
| **Is on aspirin** | [-0.50, 0.61] |  | [-0.64, 0.48] |  | [-0.22, 0.00] |  | [-0.29, -0.08] | Yes |
| **Is on clopidogrel** | [-1.38, 0.38] |  | [-1.56, 0.18] |  | [-0.30, 0.00] |  | [-0.39, -0.09] | Yes |
| **Is on metformin** | [ 0.19, 1.29] | Yes | [ 0.13, 1.22] | Yes | [-0.09, 0.09] |  | [-0.13, 0.06] |  |
| **Patient has hypertension** | [-0.39, 0.55] |  | [-0.51, 0.42] |  | [-0.09, 0.10] |  | [-0.19, 0.00] |  |
| **Townsend quartile 2** | [-1.74, 0.18] |  | [-1.74, 0.16] |  | [ 0.02, 0.44] | Yes | [ 0.02, 0.43] | Yes |
| **Townsend quartile 3** | [-0.80, 0.72] |  | [-0.82, 0.69] |  | [-0.14, 0.25] |  | [-0.13, 0.26] |  |
| **Townsend quartile 4** | [-1.21, 0.28] |  | [-1.36, 0.12] |  | [-0.11, 0.26] |  | [-0.11, 0.26] |  |
| **Townsend quartile 5** | [-1.13, 0.25] |  | [-1.26, 0.11] |  | [-0.09, 0.25] |  | [-0.14, 0.19] |  |
| **Black** | [-0.70, 1.26] |  | [-0.80, 1.14] |  | [ 0.00, 0.44] |  | [-0.07, 0.37] |  |
| **Asian** | [-1.48, 0.33] |  | [-1.54, 0.26] |  | [-0.17, 0.06] |  | [-0.15, 0.08] |  |
| **Mixed** | [ 0.40, 3.12] | Yes | [ 0.25, 2.94] | Yes | [-0.33, 0.53] |  | [-0.29, 0.56] |  |
| **Other** | [-0.50, 1.63] |  | [-0.82, 1.23] |  | [-0.03, 0.42] |  | [-0.07, 0.37] |  |

*Table S6 - Conservative 95% confidence intervals for the difference in effects between the national studies and the original study in the multivariable analyses. “GMCR” is the original published study (Greater Manchester Care Record), “N1” is the first replication study using COVID test data from the primary care data feed, and “N2” is the second replication study utilising the Second-Generation Surveillance System for the COVID test results. “T1D” are the patients with type 1 diabetes and T2D are patients with type 2 diabetes.* *“Significant” is “Yes” if the confidence interval does not span 0 and therefore the difference effect size is statistically significant between GMCR and the national study.*

| **VARIABLE** | **T1D – N1** | | **T1D – N2** | | **T2D – N1** | | **T2D – N2** | |
| --- | --- | --- | --- | --- | --- | --- | --- | --- |
|  | **CI** | **Significant** | **CI** | **Significant** | **CI** | **Significant** | **CI** | **Significant** |
| **Age** | [-0.01, 0.02] |  | [-0.01, 0.02] |  | [-0.01, -0.01] | Yes | [-0.02, -0.01] | Yes |
| **Townsend Index** | [-0.06, 0.06] |  | [-0.07, 0.04] |  | [-0.01, 0.01] |  | [-0.02, 0.00] |  |
| **Sex—male** | [-0.52, 0.27] |  | [-0.43, 0.36] |  | [0.02, 0.16] | Yes | [0.00, 0.14] |  |
| **Patient has diabetes** | [-0.28, 0.54] |  | [-0.29, 0.52] |  | [-0.24, -0.08] | Yes | [-0.28, -0.13] | Yes |
| **Patient has COPD** | [-0.91, -0.65] | Yes | [-0.91, -0.72] | Yes | [-0.62, -0.58] | Yes | [-0.68, -0.65] | Yes |
| **Latest BMI value** | [-0.11, 1.45] |  | [-0.11, 1.45] |  | [0.37, 0.56] | Yes | [0.37, 0.57] | Yes |
| **Ethnicity—Black** | [-0.58, 1.28] |  | [-0.38, 1.46] |  | [0.18, 0.54] | Yes | [0.17, 0.54] | Yes |
| **Ethnicity—Asian** | [-0.72, 0.59] |  | [-0.78, 0.50] |  | [-0.22, 0.00] |  | [-0.33, -0.11] | Yes |
| **Ethnicity—Mixed** | [0.15, 2.37] | Yes | [0.10, 2.28] | Yes | [0.11, 0.77] | Yes | [0.05, 0.70] | Yes |
| **Ethnicity—Other** | [-0.63, 1.18] |  | [-0.61, 1.15] |  | [-0.29, 0.08] |  | [-0.34, 0.02] |  |
| **Patient has hypertension** | [-0.68, 0.27] |  | [-0.72, 0.23] |  | [0.02, 0.17] | Yes | [-0.01, 0.14] |  |
