# Supplementary material for: Replicating a COVID-19 study in a national England database to assess the generalisability of research with regional electronic health record data
Source: BMJ Open. 2025 Apr 23;15(4):e093080. doi: 10.1136/bmjopen-2024-093080 (PMC12020769; doi:10.1136/bmjopen-2024-093080)
Supplement: Supplementary file 2 [file bmjopen-2024-093080.reviewer_comments.pdf]

PEER REVIEW HISTORY

BMJ Open publishes all reviews undertaken for accepted manuscripts. Reviewers are asked to complete a checklist review form and are provided with free text boxes to elaborate on their assessment. These free text comments are reproduced below.

ARTICLE DETAILS

Title (Provisional)

Replicating a COVID-19 study in a national England database to assess the generalisability of research with regional electronic health record data

Authors

Williams, Richard; Jenkins, David; Bolton, Thomas; Heald, Adrian; Mizani, Mehrdad; Sperrin, Matthew; Peek, Niels; on behalf of the, CVD-COVID-UK/COVID-IMPACT Consortium

---

VERSION 1 - REVIEW

**Reviewer**	**1**
**Name**	**Weiner , Mark**
**Affiliation**	**Weill Cornell Medicine, Population Health Sciences**
**Date**	**28-Sep-2024**
**COI**	**None**

This manuscript describes a comparison of analysis conducted with regional data versus national data, exploring the association of diabetes on hospitalization outcomes after COVID, and among patients with COVID, the impact of comorbidities and medications the impact of those covariates on outcomes. The results show reasonable concordance of the directionality of the impact of covariates, with tighter confidence intervals associated with the larger sample sizes in the national data.

The methods raise a few questions that require clarification.

First is the definition of cases and controls in the study population (page 5, lines 8-15). The text states that the cases were “patients with a diagnosis of T2D or T2D prior to a positive COVID-19 test result.” The controls were “patients with a positive COVID-19 test result but who did not have a diagnosis of diabetes on the date of their positive COVID-19 test result.” The requirement of diabetes

a positive COVID-19 test result for the cases, and the absence of diabetes

COVID-19 for the controls is an unusual distinction in the manner of identifying the presence or absence of diabetes. Someone without diabetes recorded on the date of a positive

COVID-19 test may have had a diabetes diagnosis before that date, and should be considered to have diabetes – as a matter of fact, that scenario is what defines a case patient. Please clarify the time intervals of observation for diabetes in the case and control assignments.

A second issue related to the ability to detect diabetes is the degree of health utilization prior to the date of the positive COVID-19 date. Someone with less healthcare activity has less chance to have diabetes recorded previously. Also, prior health activity may be a predictor of future hospitalizations. The manuscript does not mention nor compare this variable in the analysis.

The third issue related to the outcome itself – hospitalization within 28 days of a positive COVID-19 test. Does hospitalization on Day 0 count in this analysis? Especially early in the pandemic, many patients who were tested for COVID-19 had presented to the ED in a clinically ill state, were tested in the ED and needed emergent hospitalization. That scenario is very different from a patient with more mild illness who was tested for COVID in the ambulatory setting and may or may not have required hospitalization over the next several weeks. While the matching of cases and controls on the relative timing of COVID testing may control for these differences, it is still possible that the baseline severity of the infection may have differed in the cohorts.

Lastly, I appreciate the value of an analysis that focuses on a population with diabetes, and explores covariates that influence the risk of hospitalization in that cohort. I also recognize the value of conducting an analysis that spans patients with and without diabetes and performs a multivariate analysis that incorporates diabetes as a covariate. However, it is not clear why the first analysis of predictors of hospitalization within a cohort of T1D and T2D was conducted as a univariate analysis.

**Reviewer**	**2**
**Name**	**Cocoros, Noelle**
**Affiliation Institute,**	**Harvard Medical School and Harvard Pilgrim Health Care**
**Date**	**20-Dec-2024**
**COI**	**None**

This paper provides results from a national UK-based study intended to replicate an analysis originally conducted in a regional database. The original study was designed to assess the relationship between diabetes and hospitalization among people with COVID-19.

Abstract

• It would help readers if the Objectives also explained the specific study question related to COVID.

• The second sentence of the Results section and the Conclusions section seem to be in disagreement. Also, it would be helpful to include some basic information about the sizes of the two studies to get a sense of the difference in scale of the regional vs national studies.

Methods

• Data sources – Can you highlight major differences in the two data sources that are most relevant to the current study and its interpretation? It's not clear from the summary what key data elements are in one and not the other. A table summarizing the key parameters and data elements of both studies and where they were the same vs different would be very helpful. I see that there is a citation (#5) for another paper by this group where the "methodological" differences in the regional vs national data are discussed, but for readers to be able to assess the reproducibility of the work in this paper, more detailed information, clearly presented, is needed.

• The study design (also in the Abstract) is a retrospective cohort study. I don't think "replication" should be mentioned as that is not a specific design type.

• Key aspects of the study design need to be presented. The following are some important items that should be provided: What ages were included in the study? How was T1D and T2D identified? The manuscript implies it is based on a single diagnosis code in patient history, which would be very simple approach that could be highly misclassified. Some detail, including code lists and any differences in the two studies, should be provided. While the current focus is on replication, the main scientific results need to be able to be assessed for validity. Which types of COVID tests were included?

• For the variables not included in the replication study, how were they used in the original study? Are they "important" variables?

• Since the Townsend score results vary in the studies, and it is not a score known to at least this reviewer, it seems worth providing a summary of it in the Methods.

• What covariates were included in your fully adjusted models?

Results

• Table 1 – What proportion of each cohort had the lab tests conducted that are reported here (e.g., LDL cholesterol)? Was smoking status and BMI 100% complete? Including the applicable study periods in the table would be helpful.

• The first results really discussed are for Table S6. There is a brief reference to Tables S1-5 in the text but no information as to what they include.

• The language around statistical significance could use some editing for clarity in the text and the tables. There is significance for the actual study question (which individual variables

are associated with hospitalization) and I believe the authors also compared results across the 3 studies. It is not possible to easily follow which results are being discussed.

• I think the manuscript would benefit from discussion of the results related to risk of COVID hospitalization by diabetes status. While the replication was obviously an objective, the results of the large national analyses are interesting themselves and warrant discussion. Further, these results should be compared to other studies on this topic. While the replication is interesting, the individual studies themselves need to assessed.

Discussion

• The section on potential errors in programming for "data curation" seems surprising to this reviewer. Repeating a study does not identify potential errors per se (lines 32-35 on page 12) – instead, doing QC of a program/code can minimize errors. While I appreciate the authors' acknowledgement that human error happens, I am not sure the paragraph is particularly helpful. Instead, can the authors provide any information on how each "data curation" code is maintained? Many large data systems have robust quality checking in place for example.

• As noted above, the results from the 3 studies need to be compared to the literature. Comparing results "internally" provides only some perspective on whether the studies are reliable.

• Lines 50-51 on page 12 – Why is the prevalence of severe mental illness likely to be higher in Greater Manchester than nationally? Citations are needed. In general this paragraph is difficult to follow – there is a discussion of differences in data but I believe the authors are also saying severe mental illness prevalence may also "truly" vary by population.

VERSION 1 - AUTHOR RESPONSE

Reviewer: 1
Dr. Mark Weiner , Weill Cornell Medicine
Comments to the Author:
This manuscript describes a comparison of analysis conducted with regional data versus national data, exploring the association of diabetes on hospitalization outcomes after COVID, and among patients with COVID, the impact of comorbidities and medications the impact of those covariates on outcomes. The results show reasonable concordance of the directionality of the impact of covariates, with tighter confidence intervals associated with the larger sample sizes in the national data.

The methods raise a few questions that require clarification.

First is the definition of cases and controls in the study population (page 5, lines 8-15). The text states that the cases were "patients with a diagnosis of T2D or T2D prior to a positive COVID-19 test result." The controls were "patients with a positive COVID-19 test result but who did not have a diagnosis of diabetes on the date of their positive COVID-19 test result." The requirement of diabetes *****prior to***** a positive COVID-19 test result for the cases, and the absence of diabetes *****on the date of***** COVID-19 for the controls is an unusual

distinction in the manner of identifying the presence or absence of diabetes. Someone without diabetes recorded on the date of a positive COVID-19 test may have had a diabetes diagnosis before that date, and should be considered to have diabetes – as a matter of fact, that scenario is what defines a case patient. Please clarify the time intervals of observation for diabetes in the case and control assignments.

You are correct that the controls were patients without a diabetes diagnosis "**prior to**" (rather than "**on the date of**") their positive COVID-19 test. We have corrected this typo in the manuscript to accurately reflect this.

A second issue related to the ability to detect diabetes is the degree of health utilization prior to the date of the positive COVID-19 date. Someone with less healthcare activity has less chance to have diabetes recorded previously. Also, prior health activity may be a predictor of future hospitalizations. The manuscript does not mention nor compare this variable in the analysis.

It is true that this might have had an effect in the original study. However, as the focus of this study was to replicate exactly the previous study, it wasn't appropriate to change the analysis plan.

The third issue related to the outcome itself – hospitalization within 28 days of a positive COVID-19 test. Does hospitalization on Day 0 count in this analysis? Especially early in the pandemic, many patients who were tested for COVID-19 had presented to the ED in a clinically ill state, were tested in the ED and needed emergent hospitalization. That scenario is very different from a patient with more mild illness who was tested for COVID in the ambulatory setting and may or may not have required hospitalization over the next several weeks. While the matching of cases and controls on the relative timing of COVID testing may control for these differences, it is still possible that the baseline severity of the infection may have differed in the cohorts.

The original paper explains that the measure was actually hospitalization within 28 days of a positive test AND in the 2 days before the test to account for patients as you describe. We have updated the manuscript (section "2.6 Variables") to make this clear.

Lastly, I appreciate the value of an analysis that focuses on a population with diabetes, and explores covariates that influence the risk of hospitalization in that cohort. I also recognize the value of conducting an analysis that spans patients with and without diabetes and performs a multivariate analysis that incorporates diabetes as a covariate. However, it is not clear why the first analysis of predictors of hospitalization within a cohort of T1D and T2D was conducted as a univariate analysis.

As mentioned in section 2.7, a full explanation of the analytical approach for the original study is available in the cited paper, while this paper only contains a brief summary. Also, as this is a replication study, the task is to replicate the methods, results and findings of the original, rather than justify the original methods. However, for completeness, the original paper used a univariable analysis in order to provide a reference point that was more interpretable clinically. If the reader wanted to know if the associations were positive, negative or null, then the univariable analysis made this easier.

Reviewer: 2
Dr. Noelle Cocoros, Harvard Medical School and Harvard Pilgrim Health Care Institute,
Comments to the Author:
This paper provides results from a national UK-based study intended to replicate an analysis originally conducted in a regional database. The original study was designed to assess the relationship between diabetes and hospitalization among people with COVID-19.

Abstract
• It would help readers if the Objectives also explained the specific study question related to COVID.

We have updated the objectives accordingly.

• The second sentence of the Results section and the Conclusions section seem to be in disagreement. Also, it would be helpful to include some basic information about the sizes of the two studies to get a sense of the difference in scale of the regional vs national studies.
Methods

That is a good point. There were similarities and differences but we had highlighted the former in the results, and the latter in the conclusion. We have updated the abstract results to better reflect the similarities and differences. We have also added the population size of the two databases.

• Data sources – Can you highlight major differences in the two data sources that are most relevant to the current study and its interpretation? It's not clear from the summary what key data elements are in one and not the other. A table summarizing the key parameters and data elements of both studies and where they were the same vs different would be very helpful. I see that there is a citation (#5) for another paper by this group where the "methodological" differences in the regional vs national data are discussed, but for readers to be able to assess the reproducibility of the work in this paper, more detailed information, clearly presented, is needed.

We have provided the key differences between the 3 studies in a new table (Table 1) and referenced it in the methods.

• The study design (also in the Abstract) is a retrospective cohort study. I don't think "replication" should be mentioned as that is not a specific design type.

This is true, but "Study design" could refer to the design of the actual analysis (which in this case is a retrospective cohort study), or to the comparison between the outputs of the original study and the replication study which is the main focus of this paper. We think that describing them separately would likely lead to more reader confusion than the way it is currently presented, which seems like a good compromise.

• Key aspects of the study design need to be presented. The following are some important items that should be provided: What ages were included in the study? How was T1D and T2D identified? The manuscript implies it is based on a single diagnosis code in patient history, which would be very simple approach that could be highly misclassified. Some detail, including code lists and any differences in the two studies, should be provided. While the current focus is on replication, the main scientific results need to be able to be assessed for validity. Which types of COVID tests were included?

The original paper includes all of the above detail, and provides a link to a github repository containing the full list of clinical codes used for all parts of the analysis. Given the main focus of this paper is the replication, rather than the study itself, and given that all code lists and analysis code are identical, we have omitted these details here. However, we have updated section 2.5 of the paper:

- to make clear where the full details of the original study are available, and
- to provide the links to the github repository in this paper so people don't have to first go via the original paper.

• For the variables not included in the replication study, how were they used in the original study? Are they "important" variables?

Testosterone and sex hormone binding globulin had no effect in the original study. Low vitamin D was associated with a marginally higher incidence of hospital admission. We have updated section 2.6 to explain this.

• Since the Townsend score results vary in the studies, and it is not a score known to at least this reviewer, it seems worth providing a summary of it in the Methods.

We have added a brief summary of the Townsend score (an index of deprivation) to the methods, and provided a citation.

• What covariates were included in your fully adjusted models?

We have updated section 2.7 by replacing "other factors" with the full list of covariates.

Results

• Table 1 – What proportion of each cohort had the lab tests conducted that are reported here (e.g., LDL cholesterol)? Was smoking status and BMI 100% complete? Including the applicable study periods in the table would be helpful.

This would have been a useful comparison to make, but it was not reported on in the original study, and so was not part of this replication study. We have added the study periods to the captions of both tables. NB, due to the addition of an extra table, this comment now refers to tables 2 and 3.

• The first results really discussed are for Table S6. There is a brief reference to Tables S1-5 in the text but no information as to what they include.

They are the numbers represented in the figures. We have updated the manuscript to make that clearer.

• The language around statistical significance could use some editing for clarity in the text and the tables. There is significance for the actual study question (which individual variables are associated with hospitalization) and I believe the authors also compared results across the 3 studies. It is not possible to easily follow which results are being discussed.

You are correct that there are two instances of significance - the significance of results within each study, and also the significance of the statistical test (described in section 2.7) for assessing whether the difference in effect size between the results of the regional and national studies was statistically significant. We have updated the text to ensure that all instances of the former are fully described as: **statistically significant effect sizes in the xxx study**
And all instances of the latter are fully described as: **statistically significant difference in effect size between the two studies**

• I think the manuscript would benefit from discussion of the results related to risk of COVID hospitalization by diabetes status. While the replication was obviously an objective, the results of the large national analyses are interesting themselves and warrant discussion. Further, these results should be compared to other studies on this topic. While the replication is interesting, the individual studies themselves need to assessed.

We have already published a paper on this as referred to in section 3.1. In the original regional study we did not have enough patients with type I diabetes to draw many statistically significant conclusions. The increased power using the national data allowed us to draw conclusions which were then published. We did not publish the national results for patients with type 2 diabetes because they did not differ (as shown in this paper), and were already statistically significant in the original study.

Discussion
• The section on potential errors in programming for "data curation" seems surprising to this reviewer. Repeating a study does not identify potential errors per se (lines 32-35 on page 12) – instead, doing QC of a program/code can minimize errors. While I appreciate the authors' acknowledgement that human error happens, I am not sure the paragraph is particularly helpful. Instead, can the authors provide any information on how each "data curation" code is maintained? Many large data systems have robust quality checking in place for example.

Perhaps the paragraph was not clear enough. The intent is not to suggest that repeating a study is a way to identify errors. Instead it is to attempt to highlight any other differences between the two studies that could have led to discrepancies. The analysis code run on the curated data was identical in the two studies, so it is unlikely to have contributed to errors. However the data curation code was bespoke for each, and although subject to many checks, could have been a source of errors. We don't believe that it is, and always ensure our code is publicly available on github, but it is worth highlighting that fact. We have updated the paragraph to make this message clearer.

• As noted above, the results from the 3 studies need to be compared to the literature. Comparing results “internally” provides only some perspective on whether the studies are reliable.

As there is already a paper for the Greater Manchester data (original study), and a follow up paper for patients with type I diabetes in the national data, which both discuss and compare the effects with other literature, we felt it would be duplication if also included here. Our aim is to make the focus of this paper the ability to replicate a study, rather than a discussion of the clinical findings which are reported elsewhere.

• Lines 50-51 on page 12 – Why is the prevalence of severe mental illness likely to be higher in Greater Manchester than nationally? Citations are needed. In general this paragraph is difficult to follow – there is a discussion of differences in data but I believe the authors are also saying severe mental illness prevalence may also “truly” vary by population.

Greater Manchester has above average levels of social deprivation when compared to England. We have changed the text to make it clear that the higher deprivation in GM could mean there is indeed a higher prevalence of SMI, and provided a citation to a paper that shows the link between deprivation and SMI.

VERSION 2 - REVIEW

**Reviewer**	**1**
**Name**	**Weiner , Mark**
**Affiliation**	**Weill Cornell Medicine, Population Health Sciences**
**Date**	**16-Mar-2025**
**COI**	

I appreciate the authors responses to my earlier review and the updates they have made in response to my review and that of the other reviewer. With the purpose of the manuscript to describe the similarity of results in a national population compared to results in a regional population, I agree that some of my suggestions regarding methods would detract from the straightforward comparison.
